# Novel insights into the anatomy and histopathology of the sacroiliac joint and correlations with imaging signs of sacroiliitis in case of axial spondyloarthritis

**DOI:** 10.3389/fphys.2023.1182902

**Published:** 2023-05-11

**Authors:** Clément Prati, Thierry Lequerre, Benoît Le Goff, Bernard Cortet, Hechmi Toumi, Anne Tournadre, Hubert Marotte, Eric Lespessailles

**Affiliations:** ^1^ Department of Rheumatology, Besançon University Hospital, PEPITE EA4267, University of Franche-Comté, Besançon, France; ^2^ Department of Rheumatology, Rouen University Hospital, Inserm 1234, CIC/CRB 1404, Rouen, France; ^3^ Department of Rheumatology—CHU de Nantes, Nantes, France; ^4^ Department of Rheumatology, MABLAB ULR 4490, CHU Lille, University Lille, Lille, France; ^5^ Department of Rheumatology, Translational Medicine Research Platform, PRIMMO, University Hospital Center of Orleans, Orleans, France; ^6^ Department of Rheumatology, CHU Clermont-Ferrand, UNH UMR1019 INRAE–Université Clermont Auvergne, Clermont-Ferrand, France; ^7^ Department of Rheumatology, University Hospital of Saint Etienne, INSERM, SAINBIOSE U1059, Saint-Etienne, France

**Keywords:** spondyloarthropathy, enthesitis, ankylosing spondylitis, immunometabolism, structural progression, sacroiliac joint, MRI, sacroiliitis

## Abstract

For a better understanding of the pathophysiology of spondyloarthropathy (SpA), a detailed anatomical description of the sacroiliac joint is required because sacroiliitis is the earliest and most common sign of SpA and an essential feature for the diagnosis of ankylosing spondylitis. Beyond the anatomy, the histopathology of sacroiliac entheses and immunological mechanisms involved in sacroiliitis are crucial for a better understanding of disease causation. In this narrative review, we discuss the core anatomical, histological, and immunohistological observations involved in the development of sacroiliitis, focusing particularly on imaging-based information associated with sacroiliitis. Finally, we try to answer the question of whether at the sacroiliac joint, enthesitis precedes synovitis and subchondral bone changes in SpA.

## 1 Introduction

Axial spondyloarthritis (axSpA) includes patients with no radiographic structural changes [non-radiographic axSpA (nr-axSpA)] and radiographic structural changes [radiographic axial SpA (r-axSpA) or ankylosing spondylitis (AS)]. Known as chronic inflammatory rheumatism, it affects mainly the axial skeleton by causing inflammatory and potentially structural changes in the sacroiliac joint (SIJ), which is responsible for inflammatory back pain or buttock pain and stiffness.

A poor understanding of the etiology behind SpA has led to a wide diversity of clinicians’ views, as the exact mechanism remains debatable.

Clinically, SpA is a family of joint disorder inflammation affecting sites where the tendons or ligaments insert into the bone, known as enthesis. The major gene involved in several key pathogenic steps is HLA-B27. AS is the most characteristic form of SpA. It predictably occurs in the SIJ. This condition is often classified as SIJ dysfunction and is diagnosed as articular inflammation named sacroiliitis. However, the etiopathogenesis of AS, notably the progression from inflammation to osteoproliferation, is as yet poorly elucidated.

Although the pathological conditions causing SIJ dysfunction are consistently combinations of inflammatory and joint/bone degeneration, it is not clear whether inflammation is a consequence of a primary enthesis lesion or resultant from immunological/genetic factors. In fact, it is not known why/where inflammation begins and why/where ossification occurs. Herein, we review the literature in order to provide some insights into the following subjects: 1) normal anatomy of the SIJ and its variants; 2) histopathology and immunohistology of sacroiliitis in axSpA and correlation with imaging; 3) clinical correlation between X-ray and MRI in axSpA assessment of the primary lesions involved; 4) does enthesitis precede synovitis at the SIJ?

## 2 Normal anatomy of the sacroiliac joint and its variants

Herein, we provide a full description of the SIJ articular and ligamentous regions and innervations. Such information complemented with the joint imaging analysis is essential to understand the pathological process initiation and progression.

### 2.1 Articular sacroiliac

The articular part of the SIJ is roughly kidney-shaped, wider anteriorly than is posteriorly with an oblique orientation toward the front and top. It is bordered by a capsule. There is a joint cavity containing a minimal amount of synovial fluid with a joint capacity ranging from 0.5 to 2 mL (Vleeming et al., 2012). On standard radiographs, it is possible to examine this articular part on the frontal view with the visualization of mainly the lower and posterior parts of the joint space. The sacral part of the joint is concave and is covered with hyaline cartilage. It corresponds to the articular part of the iliac wing, which is convex and covered with fibrocartilage. The SIJ has very little mobility except during childbirth and the last three months of pregnancy. Mechanical stresses are mainly located in the anterior part of the joint, which explains the greater frequency of mechanical abnormalities at this level (Toyohara et al., 2020).

**FIGURE 1:**
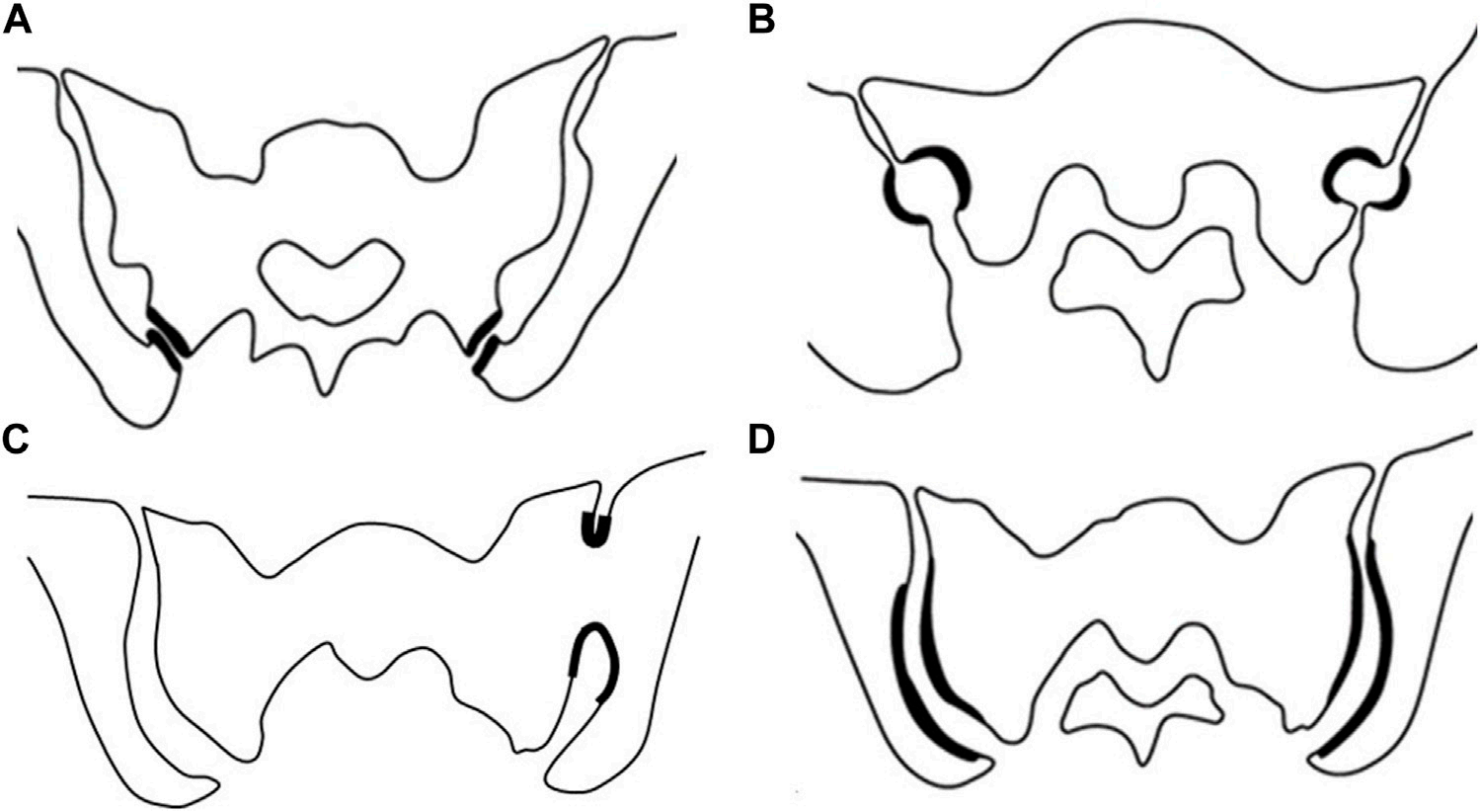
Classification of anatomical variants of the sacroiliac joint. **(A)** Accessory sacroiliac joint, **(B)** Semicircular defect, **(C)** sacroiliac fusion, **(D)** Crescent iliac bony plate [Modified from prassopoulos et al. (Prassopoulos et al. 1999)]

It is important to mention that there are many anatomical variations within the articular region of the SIJ, which may contribute to the initiation and development of degenerative abnormalities. This is also a hurdle for clinical imaging interpretation (Figure 1) (Prassopoulos et al., 1999). The accessory sacroiliac is the most common anatomic variant and has been described in 11–17% of the images performed in asymptomatic individuals (Demir et al., 2007; El Rafei et al., 2018). This accessory joint is located at the posterior and upper parts of the joint (at the level of the first and second sacral holes). It is most often unilateral and takes on the appearance of a projection of the iliac crest on the sacrum. This abnormality is more frequent with age, obesity, and repeated heavy lifting (Demir et al., 2007). It may involve a true diarthrodial joint but mostly corresponds to a fibrocartilaginous joint. The second most frequent anatomical variation is the iliosacral complex in which the iliac wing includes a convex bony protuberance that is lodged within the sacrum. Synostosis remains rare. It is often unilateral and isolated without any other abnormality, particularly of the joint space, and without condensation or erosion. In SpA, it occurs more bilaterally and late with associated lesions of established sacroiliitis, making it possible to differentiate it from congenital damage (Benz et al., 2014). Finally, the interline may be crescent-shaped with an atypical concave shape of the ilium and convexly facing the sacrum. Numerous other variations labeled “dysmorphic”, especially of the foot of the sacroiliac, are also frequently found.

Importantly, these variations of the norm could be responsible for mechanical stress on the homolateral sacroiliac and also on the contralateral sacroiliac in the case of a unilateral defect (El Rafei et al., 2018). Contralateral bone edema has been described in unilateral sacroiliac synostosis (Song et al., 2019). A recent study showed that anatomical variations such as iliosacral complexes and crescent-shaped interlineations were more frequently associated with the presence of symptoms (Ziegeler et al., 2021b). This was more prevalent in mechanically caused pain and where morphological abnormalities were present in more than 80% of the patients. Similarly, these were also associated with MRI inflammatory pieces of evidence in patients with proven SpA.

Sex and anatomical joint form can have a major impact on the development of degenerative lesions of the sacroiliac joints. The male pelvis is typically longer and narrower with a more conical shape than the female pelvis. Due to a more posterior inclination of the sacrum, the cartilaginous articular surface is located more horizontally and is generally smaller in women than in men. Anatomical variants are significantly more prevalent in women (Ziegeler et al., 2021a). Only 38% (152/401) had a typical joint form *vs.* 86% (358/417) of men (*p* < 0.001). The accessory joints were associated with the risk of dorsal sclerosis.

### 2.2 Ligamentous sacroiliac

It is important to consider the sacroiliac ligaments because they may be the source of pain in SpA *via* enthesitic involvement but probably also in mechanical pathologies related to abnormal mechanical traction. A distinction is made between the deep ligaments located on the anterior part of the joint and between the bony parts (interosseous ligament) and the superficial ligaments composed of short and long posterior sacroiliac ligaments. The anterior ligament is attached to the anterior part of the joint and capsule. It is relatively thin and presents a thin region useful for the lumbosacral plexus clinical infiltration procedure. The interosseous ligament is thicker and stronger covering the entire joint areas. The posterior short sacroiliac ligaments are stretched between the iliac crest and sacrum, whereas the posterior long sacroiliac ligament is strained between the posterosuperior iliac spine and the third and fourth sacral tubercle on the sacrum. The latter ligament plays a major role in stabilizing the joint during these movements, and hyperlaxity of this ligament can create hypermobility and pain (Eichenseer et al., 2011).

Infiltrating the ligament was more effective than intra-articular in an open/randomized series of patients with mechanical sacroiliac pain (Murakami et al., 2007). The other reason concerns their involvement during inflammatory pathological diseases. Enthesitis remains the elementary lesion common to all spondyloarthritis. The oblique coronal sequence allows the study of entheses of the pelvis and sacroiliac joints. Apart from joint involvement and bone edema, the sacroiliac ligaments should be studied carefully when reading an MRI. One study reported the sensitivity and specificity of sacroiliac ligament involvement in SpA (Jans et al., 2014). Interosseous ligament involvement was found in 7.3% of patients with a specificity of 98.9%; posterior ligament involvement was even rarer (2.8% of patients) with the same specificity of 98.9%, showing that these are very specific but not very sensitive abnormalities. In the pelvis, the most frequent abnormality is damage to the common vertebral ligament.

### 2.3 Sacroiliac joint innervation

In refractory pain joints, neurolysis of its sensory branches has been reported (Stelzer et al., 2013). In addition, it serves to better understand the atypical pseudo-sciatica-like radiations induced by its pathological involvement. The anterior part of the joint is innervated by sensory branches from the lumbosacral plexus (especially from the L4 and L5 roots) and the superior gluteal nerve. The posterior part is innervated by lateral branches of the posterior branches of the sacral nerves from S1 to S4. There are important anatomical variations of these branches that pass through or under the posterior superficial ligaments of the sacroiliac (Fortin, 2014). Neurolysis of the sensory branches of the posterior branches under imaging control has been proposed as a treatment for sacroiliac pain that is resistant to conventional therapy (Simopoulos, 2015). In open studies, it has resulted in significant improvement in this patient population.

## 3 Histopathology and immunohistology of sacroiliitis in axSpA and correlation with imaging

The histopathology of sacroiliitis is not well known due to difficulties in obtaining raw materials. In fact, only a few studies have been conducted on the histology of the SIJ in patients with axSpA, notably in early disease patients. Since the 1930s, although there have been a few isolated observations in the literature of histological studies of SIJ in SpA, it was with the work of Ball (1971) that the adventure of a histological understanding of SIJ involvement in SpA really began. We report below the histopathological and immunohistological findings concerning sacroiliitis in axial spondyloarthritis, the correlation of these lesions of sacroiliitis with imaging, their predictive value, and the kinetics of lesions of the SIJ. Table 1 summarizes the main publications in the histopathology and immunohistology assessment of the SIJ in axSpA.

**TABLE 1:** Summary of publications on the histopathology and immunohistology assessment of sacroiliac joints in axial spondyloarthritis.

Authors (references)	Year of publication	Disease	Patients (n)	Control (n)	Necropsy (n)	Biopsy (n)	Correlation with imaging
Cruickshank (Cruickshank, 1951)	1951	AS	2		1	1	
Ball (Ball, 1971)	1971	AS	8		8		X-ray
Dihlmann (Dihlmann et al., 1977)	1977	AS	1		1		
Shichikawa (Shichikawa et al., 1985)	1985	AS	6			6	
Braun (Braun et al., 1995)	1995	AS	5			5	
François (François et al., 2000)	2000	AS	12	Yes	7	5	
Bollow (Bollow, 2000)	2000	SpA	32*			12	MRI
François (Francois, 2006)	2006	AS	5	Yes		5	
Gong (Gong et al., 2012)	2012	AS	109			109	MRI
Cui (Cui et al., 2015)	2015	axSpA	36			36	X-ray and MRI
Peng (Peng et al., 2017)	2017	axSpA (r/nr)	19			19	
Wang (Wang et al., 2018)	2018	axSpA (r/nr)	193	Yes		193	

AS: ankylosing spondylitis, SpA: spondyloarthritis, r/nr axSpA: radiographic/non-radiographic axial spondyloarthritis, MRI: magnetic resonance imaging, and n: number*(18 ankylosing spondylitis, 12 undifferentiated spondyloarthropathies, and 2 psoriatic arthritis).

### 3.1 Histopathology of sacroiliitis in axSpA

One of the earliest observations was by Cruickshank (1951), who in a single biopsy from the SIJ of an AS patient, described the presence of a little granulation tissue over the surface of the hyaline cartilage, but no synovial tissue proper was seen. It has been said about Ball’s publication (1971) that “*It is therefore remarkable that the central role attributed to enthesitis in sacroiliac (SI) joints is supported by no more than three illustrations from two cases at advanced or terminal stages, neither demonstrating active disease*”. He was one of the first to use the term “enthesopathy” in SpA but never applied the term to the SIJ in fact, only to the spine. On the SIJ, he reported the case of a young patient with a short duration of symptoms (4 years), who died at the age of 22 years. SIJ serial sections histology investigation showed two main characteristics: the joint was first ankylosed by bony bridges less than 1 mm thick, spanning the periphery of the joint at intervals, and partly composed of woven bone indicating an ossification of the fibrous tissue. Second, there was continuous endochondral ossification of the articular cartilages at the iliac side (Ball, 1971). Six years later, Dihlmann et al. (1977) published the results of a single SIJ autopsy in a patient with an advanced SIJ X-ray lesion (grade III). For the first time, inflammation was observed in the synovial membrane and the bone marrow, especially near the capsular attachment zone. Similar to Ball, Dihlmann et al. noticed subchondral abnormalities, with chondroid subchondral metaplasia, causing bone and cartilage degradation. There was predominantly fibrous and fatty marrow in the subchondral bone. Over the interrupted subchondral boundary lamella, the chondroid structures expanded into the articular cartilage and replaced it. Shichikawa et al. (1985), by contrast, in a study of six patients, did not find pannus formation. Braun et al. (1995) used a new method, computed tomography–assisted biopsy of the SIJ, to improve synovial tissue harvesting that was performed in five patients with AS. In all five patients, cellular infiltrates were found invading the cartilage. François et al. (2000), for the first time, compared the microscopic features of the SIJ of 12 AS patients with seven controls (biopsies or necropsies). The lesions observed in the AS group included the loss of synovium, synovitis, major peripheral, and deeper cartilage destructions. Reduced cellularity of the bone marrow, enthesitis, chondroid differentiation, new endochondral bone formation, and fibrous and bony bridges within the synovial joints were also observed. There was no difference in the prevalence of the new bone within the ligamentous portion of the joints between the AS and control groups. Similar results were obtained for the cartilaginous bridges within the synovial joints although they developed earlier in the AS group. The initial hypothesis supporting enthesis involvement in the SIJ is not reinforced by these data.

**FIGURE 2:**
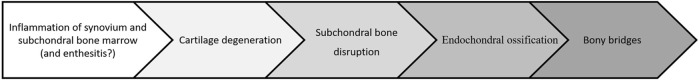
Schematization of the evolution through time of histological lesions of sacroilitis in ankylosing spondylitis.

In the 2010s, Asian teams published large series of needle biopsies of the SIJ in axSpA. The involvement of the cartilage and the subchondral bone was the most common change. These included degeneration of fibrocartilage, erosion, and ossification of the subchondral bone (Gong et al., 2012; Cui et al., 2015; Wang et al., 2018). Mononuclear cell infiltrates and fibrous tissue were also found in the bone marrow of nr-axSpA patients (Peng et al., 2017). The lesions of the SIJ in AS are summarized as proposed by Wang et al. (2018) in Table 2 and Figure 2. The prevalence of the lesions of interest is presented in Table 3.

**TABLE 2:** Different histologic lesions of sacroiliac joints seen in ankylosing spondylitis.

1) Cartilage degeneration: chondrocyte hyperplasia, hypertrophy, or focal distribution; cartilage matrix depletion or fibrosis and mucoid degeneration
2) Endochondral ossification: bone deposits on remnants of the cartilage matrix
3) Pannus formation: highly vascular granulation tissue formed from the inflamed synovium or subchondral bone marrow
4) Subchondral bone disruption: pannus invasion and destruction of the subchondral bone
5) Osteoclast activation: in areas of bone resorption at the subchondral bone endplate or the bone–cartilage interface
6) Sequestrum: a fragment of bone that has become necrotic and has separated from the normal bone structure
7) Pathologic new bone formation
8) Marrow inflammatory cell infiltration
9) Synovitis
10) Enthesitis

**TABLE 3:** Prevalence in the percentage of lesions of interest in sacroiliac joints of ankylosing.

	AS				Controls	
	Wang et al. (2018)	Cui et al. (2015)	Gong et al. (2012)	François et al. (2000)	Wang et al. (2018)	François et al. (2000)
Synovitis	35.3	58.3	33.3	100 (2 patients)	0	0
Enthesitis	29.6	25	21.1	58.3	0	9.1
Cartilage abnormalities	40.4	86.1	73.4		15.4	
Subchondral bone abnormalities	80.5	72.2	92.2		7.7	
Bone marrow infiltrate	46.9	47.2	96.3		0	

AS: ankylosing spondylitis.

### 3.2 Immunohistology of sacroiliitis in axSpA

Braun et al. (1995) were the first to perform an immunohistological analysis of the SIJ. In AS patients, pannus-like tissue was observed in five patients at the SI joint. The pannus-like tissue contained CD4^+^ (the most frequent), CD8^+^ T cells, and CD14^+^ macrophages. Cellular infiltration, notably T cells and macrophages, was also observed in another German study in early SpA (Bollow, 2000).

Another study demonstrated an accumulation of CD3^+^ T cells in the bone marrow and the synovium in early AS (Francois, 2006). The presence of macrophages in the bone marrow, the marrow spaces of the sclerotic iliac bone, and the pannus invading superficially the articular cartilage was also observed. Osteoclasts, which are CD68^+^, were seen in areas of endochondral ossification (Francois, 2006). Similar results were obtained later by Peng et al. (2017) in the bone marrow of nr-axSpA patients for CD163^+^, CD20^+^ B cell, and CD3^+^ T cells. Among the fibrous tissue specimens, all exhibited macrophage infiltrates and some exhibited B-cell infiltrates and T-cell infiltrates. Peng et al. observed that B cells were also involved in active sacroiliitis in patients with axSpA.

Dickkopf-1 (DKK1) has been shown to be one of the key factors for joint remodeling (Diarra et al., 2007). In a mice transgenic model, although blockade of DKK1 did not affect inflammatory signs of sacroiliitis, it had enhanced sacroiliac ankylosis (Uderhardt et al., 2010).

On the immunohistology of cytokines, Braun et al. (1995) first found abundant evidence for TNFα in the infiltrates and TGFß2 mRNA close to the ossified islands that suggested a multifunctional growth factor promoting new bone formation in AS. In accordance with these findings, topographical studies showed the presence of TNFα in the bone marrow of AS patients and the superficial layer of the articular cartilage. Also, TGFß1 was present in the chondroid tissue and bone cartilage, while IL10 was observed in the resorbing front of a resorption canal in the bone (Francois, 2006). When compared with controls, only patients with very early AS had increased numbers of TNFα+ cells. At the interface of the bone and cartilage in sacroiliitis patients, the subchondral microvessel density, number of CD68^+^ multinuclear osteoclasts, and the levels of VEGF, caspase-3, and MMP-3 were significantly higher than in the controls (Francois, 2006; Wang et al., 2018).

### 3.3 Correlation of histological lesions of sacroiliitis with imaging of sacroiliac joint in axSpA

Ball (1971) established the first comparison between the histology and radiology of the SIJ. He hypothesized that the radiological irregularity of the joint border (erosions) and the apparent widening can be related to endochondral ossification progression. Bone sclerosis observed on iliac radiological images was connected to the appositional deposition of the lamellar bone. Wang et al. (2018) specified the histological changes according to the X-ray grade of sacroiliitis. In grades 0–1, inflammatory cells and subchondral fibrovascular tissue were observed. Also, immunohistochemistry staining showed that CD34^+^ microvessels and CD68^+^ osteoclasts increased in the subchondral bone, yet no apparent indication on X-rays was observed. In grade 2 sacroiliitis, pannus invasion and cartilage fibrosis degeneration were observed; the cavity side pannus forms and invades into the cartilage, accompanied by endochondral ossification, and abundant CD68^+^ macrophages are expressed in the cartilage surface where the pannus has invaded. In grade 3 sacroiliitis, pannus invasion results in cartilage degeneration; abundant fibrovascular tissue forms and destroys the subchondral bone, and abundant CD68^+^ osteoclasts are expressed in areas of bone resorption (Wang et al., 2018).

MRI data have indicated more inflammatory cells in patients with few chronic lesions and those with higher inflammation scores on MRI. T cells were slightly more frequent than macrophages and B cells. This may suggest that the degree of cellularity correlates with the enhancement of the contrast agent gadolinium-DTPA detected in patients with active sacroiliitis (Bollow, 2000).

In the study by Cui et al. (2015), in 28 cases with histological changes of sacroiliitis, sacroiliitis was detected by MRI, computed tomography, and plain X-rays in 27, 22, and 28 patients, respectively, whereas in eight cases without histopathological changes of sacroiliitis, the corresponding number of patients was two, one, and one, respectively. The corresponding sensitivity was 96.4, 73.3, and 64.2%, respectively, and the corresponding specificity was 75, 87.5, and 87.5% (Cui et al., 2015). In another study, in patients with early histological sacroiliitis, MRI sensitivity and specificity of the diagnosis for early sacroiliitis were 37.7 and 100%, respectively (Gong et al., 2012).

### 3.4 Predictive histological lesions of structural progression of SIJ

To date, the histological progression of the SIJ in SpA has hardly been explored. A follow-up period of 1–13 years, with an average follow-up time of 3.6 ± 2.7 years have been performed on 98 axSpA (29 with AS and 69 nr-axSpA), which demonstrated that only 63.3% of them presented a progression of structural damage upon radiographic assessment. Multiple regression analyses showed that cartilage pannus invasion (OR 2.99, *p* = 0.010) and endochondral ossification (OR 3.97, *p* = 0.049) at baseline were the two main risk factors for radiological structural damage (Wang et al., 2018).

### 3.5 Kinetics of SIJ lesions

In their work, François et al. (2000) proposed a sequence of histologic changes that characterize sacroiliitis in AS. The developments of synovitis and myxoid bone marrow continue until the appearance of pannus and granulation tissue replaces the degenerated articular cartilage and subchondral bone. The para-articular bone becomes thicker which results from increased osteoblastic activity. Both the original and newly formed cartilages are subjected to endochondral ossification. Chondroid differentiation and fibrosis/woven bone are signs of degeneration but also of tissue repair. It has been shown that patients with “early” AS show more T cells, TNFα, and IL6, while late cases of AS show more TGFß1 (Francois, 2006).

## 4 Clinical correlation between X-ray and MRI in axSpA assessment of the primary lesions involved

### 4.1 X-ray

The classical clinical manifestation of inflammation of the SIJ is inflammatory back pain with buttock pain. However, sacroiliitis on X-rays is the result of multiple inflammation phases. Therefore at a specific time, the clinical buttock pain is not correlated with sacroiliitis grading on X-rays (Gofton et al., 1966). Furthermore, the SI reading on the X-ray is not reproducible, with high intra- and inter-reader variability mainly for grades 0 and 1.

Clinically, non-radiographic axSpA is separately classified from axSpA. In fact, they present a significant difference in the extent of structural damage (by definition) and the level of objective inflammation, as shown by the CRP values and MRI findings, and sex distribution in mainly cross-sectional studies (Sieper and van der Heijde, 2013). Yet, it is important to note that they present a noticeable similarity in clinical disease activity, diagnosis, progression, and response to TNF blockade in patients with the same disease activity levels. A study reported a 2-year follow-up period, where nr-axSpA and AS patients showed similar clinical disease courses over the 2 years with a low rate of remission in the absence of TNF blocker treatment (Poddubnyy et al., 2015).

The response rate to certolizumab (CZP) or other anti-TNF drugs is similar in both nr-axSpA and AS patients (Landewé et al., 2014). Furthermore, CZP reduces inflammation in the spine and SI joints in patients with r-axSpA and nr-axSpA (Braun et al., 2017). However, agreement between clinical remission and definitions of the absence of MRI inflammation is limited (Braun et al., 2017).

### 4.2 MRI

In a study of 41 early SpA patients, no correlation was found between patient characteristics (BASDAI, BASFI, Schober, and Physician’s Global Assessment score) and MRI abnormality scores (Puhakka, 2003). However, in a large French DESIR cohort involving 600 patients, both sites of axial pain and MRI inflammation were associated (Blachier et al., 2013).

### 4.3 Interpretation of MRI

Due to its high diagnostic performance, MRI of the SIJ is an established tool to help rheumatologists diagnose SpA. It is also the best tool to detect pathological changes in the bone marrow and in enthesitis and to identify a follow-up of the pre-structural changes on radiography and computed tomography (CT) (Oostveen et al., 1999). Nevertheless, it does not replace the global context of the demographic, clinical, and laboratory information from a given patient. Moreover, the MRI interpretation must be done very carefully and methodically to avoid conclusive suggestions to a lesion of SpA by mistake.

MRI that includes T1-weighted sequences is as accurate as CT. It detects structural changes such as bone erosions, subchondral sclerosis, and ankyloses. Fluid-sensitive sequences such as T2-weighted fat-suppressed, T2-weighted Dixon, and short-tau inversion recovery (STIR) are used to detect bone marrow edema (BME), synovitis, enthesitis, and capsulitis (Diekhoff et al., 2017).

For several years, radiologists have used new sequences with the Dixon method based on chemical shifts to perform further uniform fat suppression with fewer magnetic artifacts. From a single acquisition, suppression of the fat signal provides one image with four contrasts: i) Dixon in-phase: water and fat are imaged, like T1 and T2; ii) Dixon out: water and fat are imaged, but out of phase; iii) Dixon water: only water without fat is imaged, like FAT-SAT; and iv) Dixon fat: only fat is imaged. When used as a fat-suppressed image, the Dixon fat image can be combined with other sequences of various weightings to provide fat suppression (Table 4).

**TABLE 4:** Signal of basic lesions according to magnetic resonance imaging sequences.

	Coronal T2 DIXON «IN»	Coronal T2 DIXON « WATER»	Coronal T2 DIXON «FAT»	Coronal T2 DIXON « OPPOSITION»	
	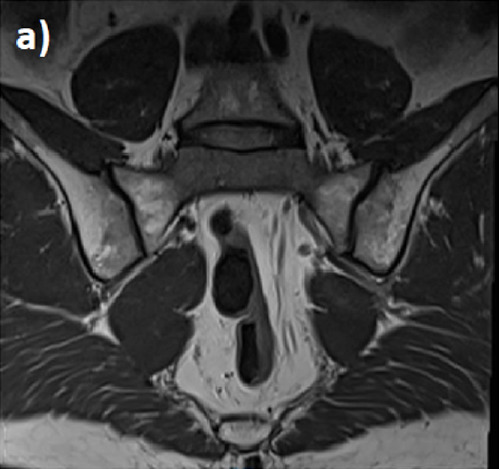	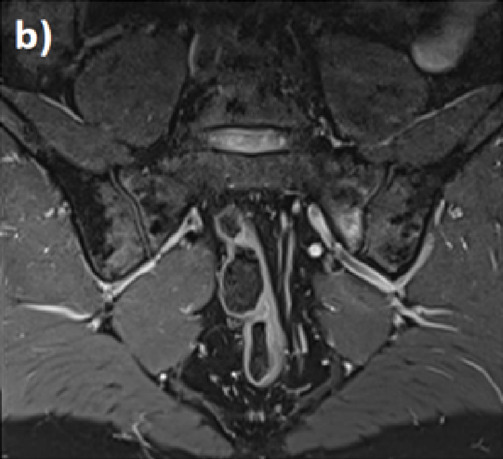	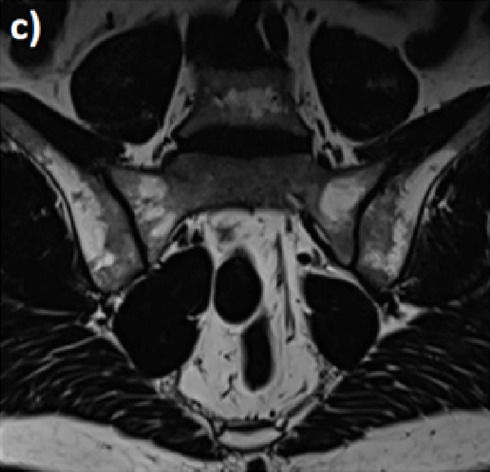	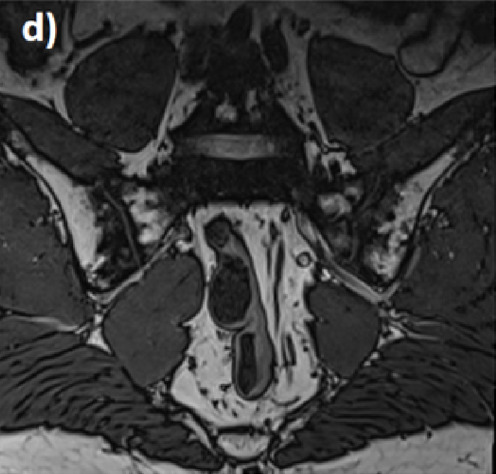	
Sequences	T1 = Dixon T1 in	T2 = Dixon T2 in	T2 fat sat = Dixon T2 water	T1 gado	T1 gado fat sat
Fat	Hyper	Hyper	Hypo	Hyper	Hypo
Water or cyst	Hypo	Hyper	Hyper	Hypo	Hypo
Edema/inflammation	Hypo	Hyper	Hyper	Hyper	Hyper

The T2 Dixon sequences: a) the Dixon T2 “IN” sequence is a usual T2 sequence without a fat signal; b) the T2 Dixon “Water” sequence is a T2 fat sat sequence without the fat signal. Only water protons are visible. This sequence is used to detect edematous signals or collections. c) The T2 Dixon “FAT” sequence is a sequence where only fat protons are visible (hypersignal) and all non-fat structures are erased. This sequence is particularly useful for bone marrow replacement images (metastases and myeloma); d) the T2 Dixon “OUT” sequence is a sequence that looks like the “IN” sequence but the outline of the organs is circled in Chinese ink.

Interpretation: the presence of an inflammatory hypersignal of the foot of the left sacroiliac joint and the anterior segment of the right sacroiliac joint. These anomalies are located in areas of mechanical stress.

The use of gadolinium contrast agents is not recommended by the EULAR for SpA (de Hooge et al., 2013).

A correct MRI SIJ lesion interpretation should consider all sequences, notably two perpendicular plane slices that include the oblique coronal plane (parallel to the posterior surface of the S2 vertebral body) and oblique axial plane. It should also involve all entire lesions (active and structural).

Moreover, lesions should highly characterize SpA and refer to it as the specific anatomical region. Any small, isolated lesion should be interpreted with caution (Maksymowych et al., 2019)**.**

Overall, we can distinguish two types of lesions: lesions related to SpA activity and those related to structural damage.

### 4.4 Lesions related to SpA activity

The main lesion that is related to SpA activity is BME alongside inflammation imaged with T1-weighted sequence bone marrow contrast enhancement (i.e., T1FS post-Gd) or T2-weighted sequence sensitive for free water (i.e., short tau inversion recovery) or T2 fat sat.

According to the ASAS group, BME is defined as a hyperintense signal on STIR images and often as a hypointense signal on T1 images. The sacral interforaminal bone marrow signal provides the sign of a normal signal in the bone marrow (Maksymowych et al., 2005). Sacroiliitis diagnosis is confirmed when the hyperintense signal is above two BME on one slice or above one BME on two consecutive slices and lesions that are highly suggestive of SpA. Lesions highly suggestive of SpA must be homogeneous, unequivocal, and multifocal, with significant size (≥1 cm), at the subchondral bone region, with poorly defined margins (Lambert et al., 2016). BME usually, but not always, appears in the posterior and inferior parts of the SIJ, where mechanical stresses are lower. But bone marrow edema changes over time and is not always specific to sacroiliitis because it can be observed in non-inflammatory conditions affecting the SIJ such as in long-distance runners and women with postpartum pain (Seven et al., 2021; Hecquet et al., 2022). All these characteristics must be finely analyzed with precision. Among non-inflammatory conditions, sex differences in pelvic anatomy should be considered. Increased pelvic tilt in females when compared to males, variations in the shape and contours of articular cartilaginous surfaces, and frequent anatomical variations in females may therefore lead to non-inflammatory changes on MRI (Jurik, 2023; Ulas et al., 2023). Moreover, bone marrow edema is frequent during pregnancy and also during postpartum, which is still present in more than half of patients at 12 months postpartum (Kiil et al., 2023).

Other lesions can be visualized such as capsulitis (amplified signal on STIR and/or T1FS post-Gd), alongside enthesitis. These are detected as high signals in the bone marrow and/or soft tissue on STIR, a bright signal in the joint space on STIR images, and an increased signal on STIR visualized after contrast medium administration, resulting from increased signals in the joint space of the cartilaginous portion of the sacroiliac joint and inflammation at the site of erosion (Maksymowych et al., 2019).

The presence of only synovitis, enthesitis, or capsulitis without BME does not permit a positive MRI-SI diagnosis (Rudwaleit et al., 2009). In fact, without BME MRI signs, the occurrence of structural lesions such as fat metaplasia, sclerosis, erosion, or ankylosis is not sufficient to fully confirm an effective sacroiliitis on MRI (Lambert et al., 2016).

In patients with SpA, BME can occur in all quadrants and all three sections of the joint and sometimes bilaterally (Seven et al., 2021).

### 4.5 Lesions related to structural damage

Structural damage at the SIJ includes erosions, osteophytes, fat lesions, ankylosis, and sclerosis.

Erosions are defined as bony defects in the subchondral bone. It corresponds to a full-thickness loss of the dark appearance of the subchondral cortex at its expected location. It is also accompanied by a loss of signal on a T1W non–fat-suppressed sequence when compared with the normal bright appearance of the adjacent bone marrow (Maksymowych et al., 2019). They are specific to SpA, mainly iliac, and often bilateral (Seven et al., 2021).

Fat metaplasia has a homogeneous bright signal seen on a T1W non–fat-suppressed sequence. It is found in the subchondral bone region, brighter than the regular bone marrow, and sharply distinct along its non-articular border with the normal bone marrow (Maksymowych et al., 2019). They are mostly present in axSpA. They appear in all quadrants, but often bilaterally in sacral quadrants (Seven et al., 2021)**.**

A bright signal on the T1-weighted with an intensity greater than that of the normal bone marrow observed in erosion or confluent erosions region suggests fat metaplasia in an erosion cavity or backfill. It is often accompanied by a total loss of the dark facade of the subchondral cortex (a reparative process resembling the transformation of subchondral BME into fat metaplasia) and is clearly demarcated from the adjacent bone marrow by an irregular band of dark signal reflecting sclerosis at the border of the original erosion (Maksymowych et al., 2019). This lesion characterizes a structural lesion resulting from the resolution of inflammation in an erosion cavity.

Sclerosis appears as an area with a hypointense signal in all sequences (T1, STIR, T1 post-Gd sequences, and T2 Dixon), without signal enhancement after contrast medium administration. Sclerosis attributable to SpA should extend at least 5 mm from the SI joint space since small areas of periarticular sclerosis can be observed in healthy subjects (physiological sclerosis) (Rudwaleit et al., 2009).

Ankylosis matches with an abnormally bright signal on a T1W non–fat-suppressed sequence together with a similar signal intensity to the bone marrow at the expected SIJ space location. It bridges the joint resulting in a continuous bone marrow signal between the ilium and sacrum. It is also associated with full-thickness loss of the dark appearance on both sides of the joint at the subchondral cortex (Maksymowych et al., 2019). Ankylosis is confirmed when the bright bone marrow signal across the joint space is continuous.

Bone buds include images with an abnormally bright signal on a non–fat-suppressed sequence T1W. The signal has a similar intensity to that of the bone marrow, located at the expected region of the SIJ space and continuous with the subchondral bone of either the ilium or sacrum but not both. A full-thickness loss of the dark appearance of the subchondral cortex on the corresponding side of the joint is also observed in the same region (Maksymowych et al., 2019).

Backfill and ankylosis may be considered pathognomonic for axSpA. Yet, they are not relevant for improving early diagnosis because of their late occurrence diagnosis (Seven et al., 2021).

As indicated above, all lesions with their location and distribution in the SIJ and their combination must be taken into account for MRI interpretation. The different lesion assessment combinations advance MRI diagnostic capacities. The combination of BME, fat lesion, erosion, and sclerosis, associated two by two, particularly when located centrally or posteriorly, is diagnostically important for SpA (Seven et al., 2020).

## 5 Does enthesis precede synovitis at the sacroiliac joint?

It has been demonstrated that SpA is associated with primary enthesitis (McGonagle et al., 2002; Benjamin et al., 2007; Benjamin and McGonagle, 2007). Initiated enthesis damage extends consecutively to the synovium and adjacent bone. The concept of an enthesis organ as labeled by Benjamin et al. (2007) is of general significance in understanding the anatomopathology assessment of these structures in the pathogenesis of SpA. Furthermore, it is important to consider the synovio-entheseal complex similarly to that of an enthesis organ. Its zone is beyond the interface between the bone and ligament, and also the surrounding tissues, which include periosteal fibrocartilage, bursa, fat, and synovium. Enthesis organs are sensitive to microdamage, and the presence of HLA-B27 may be related to synovium and bone marrow immune cells and increase immune responses (McGonagle et al., 2002). In early enthesitis, the connection of HLA-B27 with bone pathology requires further research and may contribute to a better understanding of the pathogenesis of SpA. In studies comparing patients with SpA-associated disease to mechanically induced disease, it has been shown that BME is more severe in HLA-B27-positive patients, confirming the role of HLA-B27 in the inflammation process resulting from mechanical stress (Benjamin et al., 2007; Benjamin and McGonagle, 2007).

Several authors have however challenged the involvement of the enthesis organ in the SIJ in AS. They point out that enthesis is neither the predominant process nor the initial pathologic process in sacroiliitis of AS (François et al., 2000). Two other explanations have been proposed: 1) joint destruction is caused by synovitis and subchondral bone marrow alterations and 2) ankylosis is triggered by the abnormal form of chondroid metaplasia (François et al., 2000). It has been suggested that the involvement of enthesitis cannot be justified because the histologic defects are located at the cartilaginous level rather than in the entheses portions of the SIJ (François et al., 2000). Likewise, it has been proposed that the two major radiologic signs of subchondral sacroiliitis are bone erosions and sclerosis of the adjacent bone (Sudoł-Szopinska and Urbanik, 2013), and not every SIJ abnormality is an inflammatory sacroiliitis (Tsoi et al., 2019). It was shown, however, that ultrasonography in the B mode associated with power Doppler permits peripheral enthesitis detection and abnormal vascularization in all SpA subtypes that including SpA when compared with diagnoses in control subjects (D’Agostino et al., 2003).

It is beyond the scope of this study to report the utility of ultrasound in the assessment of sacroiliitis (Rosa et al., 2019; Falsetti et al., 2021). Ultrasound (US) is not recommended among imaging techniques in the diagnosis or management of sacroiliitis in patients with SpA and can only depict a limited portion of the posterior joint space (Mandl et al., 2015; Gutierrez et al., 2018). Its precise role in the assessment of sacroiliitis is still debated (Gutierrez and Pineda, 2017).

However, in patients with limitations or contraindications to MRI, ultrasound is an alternative and there is no doubt of its value in guiding sacroiliac joint injections (Al Khayyat et al., 2022). By contrast, it can show the posterior ligaments, but no report of pathological changes in these structures has been published so far (Le Goff et al., 2011).

Moreover, in the early stages of sacroiliitis, which was one of the topics of the present article, it is difficult to adequately visualize the lower third of the sacroiliac joint, a site where the inflammatory process is supposed to begin (Zacchino et al., 2013).

## 6 Conclusion

Sacroiliitis of AS involves syndromes at the entheses, synovium, cartilage, and underlying subchondral bone. At the developed stage, the clinical symptoms of AS are clearly defined, whereas, at the early stages, the initial events are much more complex. Each of the enthesis organs, that is, the cartilage and fibrocartilage, and the related subchondral bone are connected and could potentially contribute to the disease process; at this research stage, no single organ can be designated as the unique cause of AS.

Further research with higher numbers of biopsy samples from early cases is required to define the signaling pathways responsible for the pathology initiation and draw definitive conclusions about the histopathogenesis.

## Data Availability

The original contributions presented in the study are included in the article/Supplementary Material. Further inquiries can be directed to the corresponding author.

## Author’s Note

This publication follows discussions and interactions that occurred in the context of a Novartis advisory board. However, the decision to produce a scientific communication was taken only by the authors and carried out without any Novartis support or involvement.
